# Infusion of Host-Derived Unlicensed NK Cells Improves Donor Engraftment in Non-Myeloablative Allogeneic Hematopoietic Cell Transplantation

**DOI:** 10.3389/fimmu.2020.614250

**Published:** 2021-01-07

**Authors:** Maite Alvarez, Antonio Pierini, Federico Simonetta, Jeanette Baker, Kristina Maas-Bauer, Toshihito Hirai, Robert S. Negrin

**Affiliations:** ^1^ Blood and Marrow Transplantation, Stanford University School of Medicine, Stanford, CA, United States; ^2^ Program for Immunology and Immunotherapy Department, Center for Applied Medical research (CIMA), Universidad de Navarra, Pamplona, Spain; ^3^ Navarra Institute for Health Research (IdiSNA), Pamplona, Spain; ^4^ Centro de Investigación Biomédica en Red de Cáncer (CIBERONC), Madrid, Spain

**Keywords:** Unlicensed NK, allogeneic hematopoietic cell transplantation, chimeras, engraftment, non-myeloablative conditioning regimen

## Abstract

Allogeneic hematopoietic cell transplantation (allo-HCT) is an efficacious and frequently the only treatment option for some hematological malignances. However, it often faces severe morbidities and/or mortalities due to graft *versus* host disease, and the severity of the conditioning regiment needed, that result in toxicity-related issues poorly tolerable for some patients. These shortcomings have led to the development of less aggressive alternatives like non-myeloablative (NMAC) or reduced-intensity conditioning regiments (RIC). However, these approaches tend to have an increase of cancer relapse and limited persistence of donor-specific chimerism. Thus, strategies that lead towards an accelerated and more durable donor engraftment are still needed. Here, we took advantage of the ability of host-derived unlicensed NK (UnLicNK) cells to favor donor cell engraftment during myeloablative allo-HCT, and evaluated if the adoptive transfer of this cell type can improve donor chimerism in NAMC settings. Indeed, the infusion of these cells significantly increased mixed chimerism in a sublethal allo-HCT mouse model, resulting in a more sustainable donor cell engraftment when compared to the administration of licensed NK cells or HCT controls. We observed an overall increase in the total number and proportion of donor B, NK and myeloid cells after UnLicNK cell infusion. Additionally, the extension and durability of donor chimerism was similar to the one obtained after the tolerogenic Tregs infusion. These results serve as the needed bases for the implementation of the adoptive transfer of UnLicNK cells to upgrade NMAC protocols and enhance allogeneic engraftment during HCT.

## Introduction

Hematopoietic cell transplantation (HCT) is a preferred option for the treatment of a number of malignant and non-malignant hematological diseases as well as severe combined immune deficiencies (1). In order to achieve maximal donor stem cell engraftment, many transplantation protocols have involved the administration of myeloablative conditioning regimens (MAC) through total body irradiation or high dose chemotherapy, which eliminates the host’s immune system and allows for donor hematopoietic stem cell (HSC) engraftment. However, at the initial stages after transplantation patients are susceptible to cancer relapse and opportunistic infections due to the lack of immune defense. Additionally, the development of graft *versus* host disease (GvHD) following allogeneic HCT can result in significant mortality risk despite being associated with anti-tumor responses (1). Furthermore, aggressive conditioning regimens are associated with high toxicities and treatment-related mortality (TRM) making these therapies inaccessible to elderly patients and patients with poor performance status and impaired organ function (2). Interestingly, current protocols that use non-myeloablative (NMAC) or reduced-intensity conditioning regiments (RIC), which are less toxic, are able to accomplish cell engraftment and graft versus leukemia (GvL) effects with significant reduction of TRM when compared to MAC (3). These regimens have been associated with mixed hematopoietic chimerism in the recipients. The extension and durability of these allogenic mixed chimeras are important to establish a long-term allograft acceptance with minimal or absent immunosuppression in an effort to induce transplantation tolerance (4). Unfortunately, increased cancer relapse rates, attaining durable donor-specific chimerism, and immune tolerance towards donor antigens are still major concerns in HCT when NMAC is used.

Current approaches to improve HCT tolerance are achieved through the adoptive transfer of immune cells with both tolerogenic and/or effector functions. Our group and many others have utilized the suppressor properties of regulatory T cells (Tregs) to prevent GvHD and improve tolerance to donor HSC engraftment (5–9). Similarly, NK cells provide a protective effect in allogeneic HCT outcome (1, 10). Indeed, NK cells can suppress GvHD due to the elimination of alloreactive T cells and/or antigen presenting cells (APC) preventing the T cell immune barrier to allogeneic HCT engraftment (10–12). However, host NK cells also play a critical role in breaking immune tolerance to allogeneic cells. Host-type licensed NK (LicNK) cells, those NK cells expressing inhibitory receptors that recognize self-MHC, are preferentially involved in the rejection of allogeneic HSC, unlike unlicensed NK cells (UnLicNK) (13). In contrast, new evidence supports the use of activated UnLicNK cells as a means to increase donor specific tolerance and engraftment when donor MHC class I (MHCI) interacts with their inhibitory receptor, indicating a unique function of UnLicNK cells (14). In this study, we exploited the ability of UnLicNK cells to enhance donor-specific mixed chimerism prior to NMAC allogenic HCT in order to achieve more rapid and sustained chimerism.

## Material and Methods

### Mice

C57BL/6 (H-2^b^) and BALB/c (H-2^d^) mice were purchased from Jackson Laboratories (Sacramento, CA). CD45.1^+^ congenic mice were bred in our animal facility. Female mice were used at 8-12 weeks of age and housed under specific pathogen-free conditions. All animal protocols were approved by the IACUC at Stanford University.

### Purification of NK Cells and Tregs

For NK cells, single cell suspensions from spleen and BM cells were collected from C57BL/6 mice and T-cell were depleted using EasySep™ mouse CD90 selection kit according to manufacturer’s instructions (StemCell Technology, Vancouver). Cells were then cultured in RPMI complete media at 37°C with 5% CO_2_ and supplemented with 1,000 IU/ml recombinant human IL-2 (IL-2) from NCI repository (Frederick, MD). As previously described, adherent NK cells were collected on day 5 and stained for CD45.1, TCRβ, CD122, Ly49G2, and Ly49C/I. Gated CD45.1^+^TCRβ^-^CD122^+^ cells were flow sorted by FACS Aria II (BD) for total NK, licensed or unlicensed NK cells based on their Ly49G2 and Ly49C/I expression profile according to the mouse strain. CD4^+^CD25^high^ Tregs were isolated as previously described (15).

### Transplantation

One million sorted *ex vivo* IL-2 expanded host-type NK cells (Ly49C/I^+/-^Ly49G2^+/-^), LicNK (Ly49C/I^+^Ly49G2^-^) or UnLicNK (Ly49C/I^-^Ly49G2^+^) cells, obtained as previously described (14) (see **Supplemental Material** for a detailed description), were intravenously injected into C57BL/6 mice that received sublethal irradiation (700cGy). Mice were treated with low doses (5x10^4^ IU) of IL-2 or PBS intraperitoneally (ip) for 7 days after irradiation to maintain and/or activate NK and Tregs respectively. Two days later, mice received an intravenous injection of 10 million NK and T cell depleted (NTCD) BALB/c donor-derived BM cells (negative selection of anti-CD4, anti-CD8, and anti-CD49b microbeads kits from Miltenyi Biotec). After transplantation, cell chimerism in peripheral blood (PB) was calculated according to the frequencies of donor-type MHCI. In some experiments, splenocytes were collected at day 28 post-HCT to evaluate immune parameters by flow cytometry.

Similarly, one million host-type sorted NK cells (total NK: Ly49C/I^+/-^Ly49G^+/-^; LicNK: Ly49C/I^-^Ly49G^+^; or UnLicNK: Ly49C/I^+^Ly49G^-^) and/or half a million host-type sorted Tregs were intravenously injected into BALB/c mice that received sublethal irradiation (550cGy). Mice were treated with low doses (5x10^4^ IU) of IL-2 or PBS intraperitoneally (ip) for 7 days after irradiation. Two days after NMAC regimen, mice received an intravenous injection of 5 million NTCD C57BL/6 donor-derived BM cells. In this set of experiments, to select host-type NK cells from BALB/c mice, anti-mouse Ly49G clone 4T8 was used.

The irradiation dose was chosen based on the lethal dose of total body radiation without BMC rescue for each of the strains. For C57BL/6 this dose is 950cGy whereas for BALB/c the dose is 800cGy. To accomplish a non-myeloablative regimen a 150cGy reduction from the TBI tolerated was used.

### Flow Cytometry Analysis

Antibody staining of peripheral blood lymphocytes (PBL) or single-cell suspensions from spleen was performed as previously described (16). Briefly, cells were pre-incubated with Fc-block (anti-CD16/32) 10 min at 4C to prevent unspecific binding, followed by incubations with surface mAbs for 20 min at 4C. Cells were then washed with staining buffer (PBS supplemented with 2% FBS). For transcription factors, the Foxp3/transcription factor staining buffer set was used following manufacturer’s instructions (ThermoFisher). Stained cells were analyzed with an LSRII cytometer (Becton Dickinson, San Jose, CA). Fluorescence minus one (FMO) or biological comparison controls were used for cell analysis. See **Supplementary Table 1** for a detailed description of antibodies used. Data analysis was performed using FlowJo software (TreeStar).

### Stimulation

For NK cell subset stimulation, a million cells were stimulated with anti-NK1.1 (clone PK136) coated 24-well plates for 4 h at 37°C with 5% CO_2_. For analysis of NK and CD8 T cell function 28 days after HCT, 2 million splenocytes were stimulated for 4 h at 37°C with 5% CO_2_ with 160 ηg/ml phorbol 12-myristate 12-acetate (PMA) and 1.6μg/ml ionomycin (Io) in complete media. After stimulation, cells were collected and stained for IFNγ using BD Cytodix/Cytoperm Fixation/permeabilization kit following manufacturer’ instructions.

### Serum Cytokine Analysis

The level of cytokines in the serum collected at day 28 post-HCT was measured using a multiplex assay (Luminex, Life-Technologies^©^) with the Th1/Th2/Th9/Th17/Th22/Treg Cytokine 17-Plex Mouse ProcartaPlex™ Panel (ThermoFisher) following manufacturer’s instructions.

### Statistical Analysis

Each experiment was performed at least 2 times with three to five mice per group. Student’s two-tailed t-test, one-way ANOVA (Bonferroni post-test analysis) or two-way ANOVA (Bonferroni post-test analysis) were used when appropriate to determine statistical significance (Graphpad Prism 4, La Jolla, CA). P-values were considered statistically significant when p<0.05.

## Results

### Infusion of Host-Derived Unlicensed NK Cells Improves Allogeneic BMC Engraftment After Non-Myeloablative Hematopoietic Cell Transplantation

**Figure 1:**
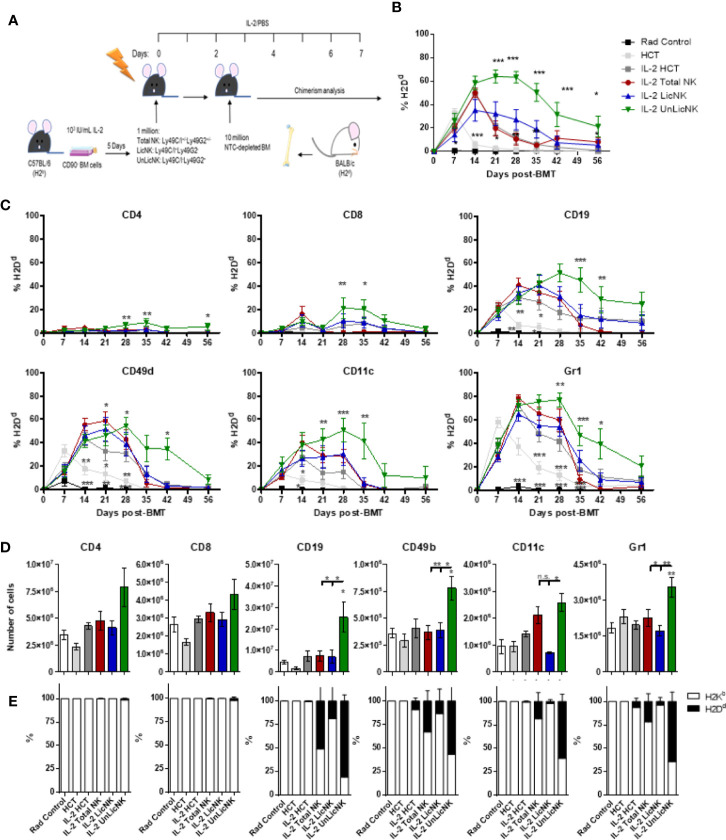
Infusion of host type activated UnLicNK cells improves donor cell engraftment after NMAC allogeneic HCT. Sublethally irradiated C57BL/6 mice received a million *ex vivo* IL-2 expanded total NK cells (Ly49C/I^+/-^Ly49G2^+/-^), LicNK cells (Ly49C/I^+^Ly49G2^-^), UnLicNK (Ly49C/I^-^Ly49G2^+^) cells or PBS followed by 10 million NTCD-BMC 2 days later. Mice were treated with IL-2 (5x10^4^ IU/ml) or PBS for seven consecutive days following NK cell transfer. **(A)** Schematic representation of NMA HCT regimen. **(B)** Percentage of total H2D^d+^ cells at each time point after NMAC HCT is shown for gated CD4 (CD45.2^+^TCRβ^+^CD4^+^), CD8 (CD45.2^+^TCRβ^+^CD8^+^), CD19 (CD45.2^+^TCRβ^-^CD19^+^), CD49b (CD45.2^+^TCRβ^-^CD49b^+^), CD11c (CD45.2^+^ TCRβ ^-^CD19^-^CD11c^+^), and Gr1 (CD45.2^+^ TCRβ ^-^CD19^-^CD11b^+^Gr1b^+^) -positive cells. **(C)** Percentage of donor H2D^d+^ cells among CD4^+^, CD8^+^, CD19^+^, CD3^-^CD49b^+^, CD11c^+^, or Gr1^+^ cells is shown. **(D)** Total number of CD4^+^, CD8^+^, CD19^+^, CD3^-^CD49b^+^, CD11c^+^, and Gr1^+^ cells is shown at day 28 post-HCT in the spleen. **(E)** Proportion of host (H2K^b^) and donor (H2D^d^) is shown for each cell type in the spleen at day 28 post-HCT. Data is representative of at least two independent experiments with n=3–5 per group (mean ± SEM). One-way ANOVA or Two-Way ANOVA was used to assess significance. Significant differences are displayed for comparisons with the IL-2 HCT control group (*p<0.05, **p<0.01, ***p<0.001).

To evaluate the potency of total-, Lic-, and UnLic-NK cells on alloengraftment, we injected each NK cell subset into sublethally irradiated H2b^+^ recipient mice, and IL-2 was administered daily for 7 days to maintain the transferred NK cells. Two days later, NTCD-BMCs obtained from H2d^+^ donor mice were injected, and PB chimerism was assessed by flow cytometry every 7 days (**Figure 1A**). IL-2 injection alone delayed donor hematopoietic cell rejection compared to PBS injection alone (**Figure 1B**), as expected given the ability of low dose of IL-2 to selectively activate and expand host Tregs (5, 17). When host-type IL-2 activated UnLicNK cells (Ly49C/I^-^Ly49G2^+^) were administered in NMAC HCT settings, we observed the highest and most sustained engraftment of H2Dd^+^ donor cells when compared to administration of total NK cells (Ly49C/I^+/-^Ly49G2^+/-^), LicNK cells (Ly49C/I^+^Ly49G2^-^), IL-2 treated or PBS HCT controls (**Figures 1A, B**). From all the immune cells analyzed, a higher donor cell engraftment (or donor cell chimerism) was particularly observed for B, NK, CD11c^+^ DC, and Gr1^+^ myeloid cells, while just a small portion of donor T cells were present (**Figure 1C**). Despite this increase in donor cells, the total percentage of each cell type was maintained along all the groups except for Gr1^+^ myeloid cells that were at higher levels in UnLic NK cell group for up to 2 months post-HCT (**Supplementary Figure 1**). However, the analysis of the immune cell compartment from the spleens collected at the peak of engraftment (day 28 post-HCT) showed a significant increase in the total number of B, NK, CD11c^+^, and Gr1^+^ cells by UnLicNK cell infusion, with a higher percentage of H2Dd expression in this group when compared to the other groups (**Figures 1D, E**). The improvement of donor cell engraftment was not due to phenotypic and functional differences between activated LicNK or UnLicNK cells as no major differences were reported in the expression of NK cell receptors, activating transcription factors, proliferation capacities or function (**Supplementary Figure 2**) (18). Importantly, as previously observed (19), infused CD45.1^+^ NK cells were not detected shortly after IL-2 treatment cessation due to the well-known contraction phenomenon (16), suggesting that UnLicNK affects HCT during early stages of reconstitution (data not shown).

As previously demonstrated, UnLicNK cells favored allogeneic engraftment through the production of GM-CSF with elevated levels of GM-CSF found in the co-cultures of sorted UnLicNK cells with allogeneic BMC, but not with syngeneic BMC, when compared with LicNK cells (**Supplementary Figure 3A**) **(** 14). Consequently, allogeneic BMCs derived from co-cultures with UnLicNK cells displayed higher hematopoietic potential, which was abrogated by anti-GM-CSF treatment (**Supplementary Figure 3B**). In contrast, no differences were found regarding the levels of the inflammatory cytokines IFNγ and TNFα in the supernatant of allogeneic BMC with LicNK or UnLicNK cells (**Supplementary Figures 3C, D**). We also evaluated the immunosuppressor cytokine TGFβ, but no differences were found (**Supplementary Figure 3E**).

**Figure 2:**
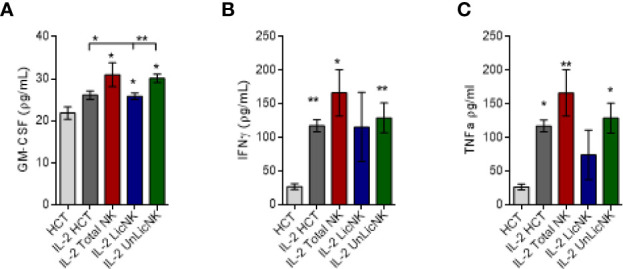
Analysis of GM-CSF, IFNγ and TNFα after NMAC HCT. Serums were collected form mice receiving NMAC HCT treatment regimen depicted in **Figure 1A**. and the cytokine levels were measured by a multiplex assay. **(A–C)** Levels of GM-CSF **(A)**, IFNγ **(B)**, and TNFα **(C)** in the serum. Data is representative of two independent experiments with n=3–5 per group (mean ± SEM). One-way ANOVA or student t-test were used to assess significance. (*p<0.05, **p<0.01, ***p<0.001, n.s, no significance).

Similarly, the analysis of GM-CSF in the serum of mice treated with NMAC HCT showed a higher presence of GM-CSF in those groups that received total or UnLicNK cells, whereas no differences were found for IFNγ and TNFα between the IL-2 treated NMCA HCT groups (**Figure 2**). The differences in GM-CSF levels between groups during the *in vivo* experiment were not very striking if compared to the *in vitro* experiments, which is likely attributed to the reduced half-life of GM-CSF (6–9 h) and the usage of GM-CSF *in vivo* for BM engraftment. Nevertheless, taking together, these results suggest that GM-CSF is also involved in the mechanism by which UnLicNK cells regulate allogeneic BMC engraftment.

### Unlicensed NK Cells Favors the Homeostatic Reconstitution of Both Host-Derived and Donor-Derived Immune Cells

**Figure 3:**
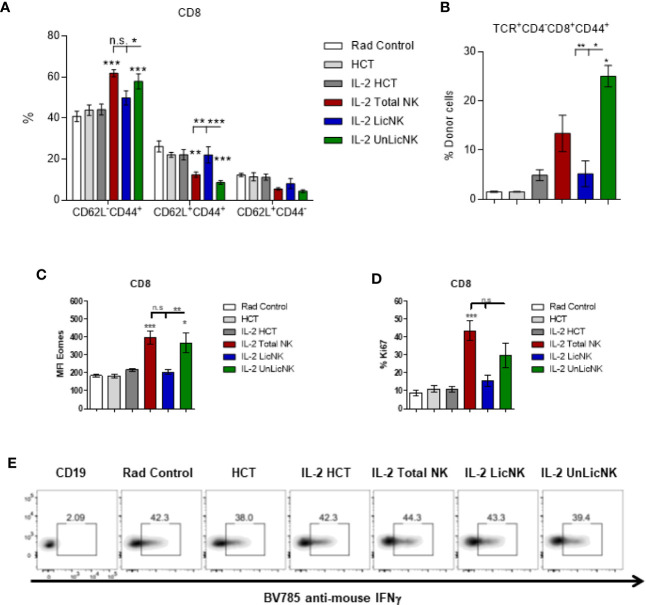
The treatment with UnLicNK cells causes an increase of donor-derived effector-memory CD8 T cells. Splenocytes were collected at day 28 post-HCT and phenotypic analysis was performed by flow cytometry. **(A)** Percentage of effector memory (CD62L^-^CD44^+^), central memory (CD62L^+^CD44^+^), and naïve (CD62L^+^CD44^-^) cell subsets for CD3^+^CD8^+^ T cells is shown. **(B)** Percentage of H2Dd^+^ cells within the CD8 T effector memory subset is shown. **(C)** MFI expression of the activating transcription factor eomes is shown for total (H2b/d) CD3^+^CD8^+^ T cells. **(D)** Percentage of Ki67 for gated total CD8 T cells is shown. **(E)** Representative dot-plots of IFNγ^+^ cells on gated total CD8 T cells after stimulation with PMA/Io. Data is representative of two independent experiments with n=3–5 per group (mean ± SEM). One-way ANOVA or Two-Way ANOVA was used to assess significance (*p<0.05, **p<0.01, ***p<0.001, n.s, no significance).

**Figure 4:**
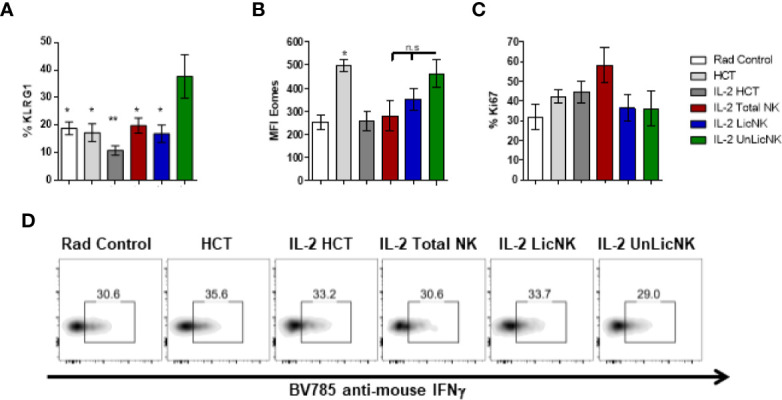
NK cell activation status during NMAC HCT. **(A)** The percentage of KLRG1 is shown for gated total (H2b/d) NK cells (CD3^-^CD49b^+^). **(B)** MFI expression of the activating transcription factor eomes is shown for total NK cells **(D)**. **(C)** Percentage of Ki67 for gated total NK cells is shown. **(D)** Representative dot-plots of IFNγ^+^ cells on gated total NK cells after stimulation with anti-NK1.1. Data is representative of two independent experiments with n=3–5 per group (mean ± SEM). One-way ANOVA was used to assess significance. (*p<0.05, **p<0.01, n.s, no significance).

Unlike the other cell types, T cells only showed a slight, but not significant, increase of total numbers and practically all the cells were host-derived (**Figure 1**). No major differences were found in the CD4 T cell compartment in terms of numbers and activation phenotype (**Supplementary Figure 4**). However, a significant increase in CD8^+^ T cells with an effector memory profile (CD62L^-^CD44^+^) was observed in the mice that received UnLicNK cells or total NK cells compared to IL-2 HCT controls (**Figure 3A**), a subset which reconstitution has been associated to control of tumor growth (20). Interestingly, the expression of H2Dd was much higher within this T cell subset (**Figure 3B**) when compared to the overall CD8 T cell population (**Figure 1D**). According to a larger activation stage, we observed an increase in the expression of the activating transcription factor eomesodermin (**Figure 3C**) and the proliferative maker Ki67 (**Figure 3D**) in the total (H2d/b^+^) CD8 T cells of mice receiving total or UnLicNK cells when compared to IL-2, PBS HCT, and rad controls. The functional stage of these cells, measured by IFNγ production, was conserved along the groups (**Figure 3E**). In contrast, donor NK cell reconstitution and expansion were favored by the treatment with UnLicNK cells (**Figures 1C, D**). The activating phenotype, and the capacity to proliferate and respond to stimuli, measured by IFNγ production, of total NK cells were preserved in all HCT conditions (**Figures 4A–D**). From all the parameters analyzed only KLRG1 expression was upregulated on NK cells from mice that received UnLicNK cells and a mild, but not significant increase of Eomes was also observed (**Figures 4A, B**) that could indicate an improvement in the activations status. Additionally, the levels of IFNγ were significantly higher in the co-cultures of sorted UnLicNK cells with syngeneic BMC when compared to LicNK cells, suggesting a superior effect against cells expressing self-MHCI (**Supplementary Figure 3C**).

**Figure 5:**
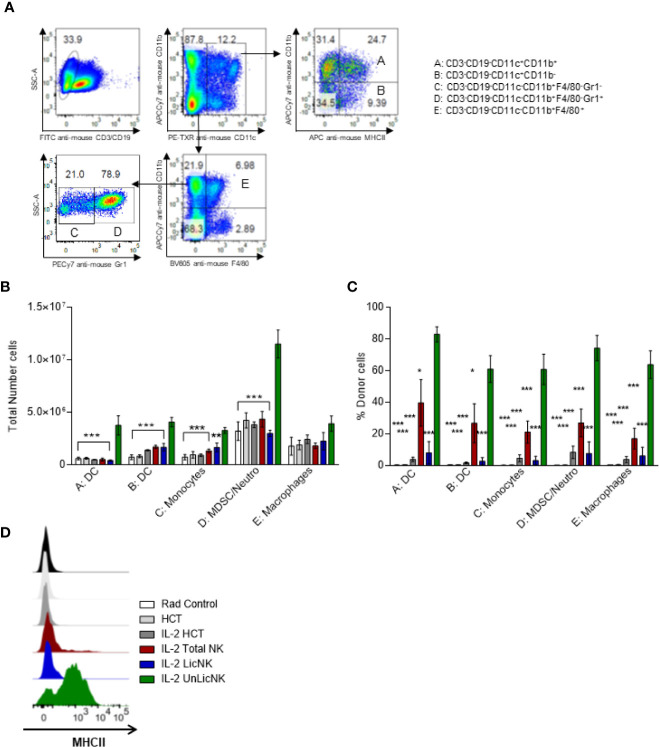
Impact of the early presence of host type activated UnLicNK cells after NMAC allogeneic HCT for myeloid cell reconstitution. **(A)** Representative Pseudo-color-plots for the gating strategy to differentiate the myeloid cell compartment. **(B)** The total number of myeloid-derived DC (CD3^-^CD19^-^CD11c^+^CD11b^+^), CD11b^-^ DC (CD3^-^CD19^-^CD11c^+^CD11b^-^), monocytes (CD3^-^CD19^-^CD11c^-^CD11b^+^F4/80^-^Gr1^-^), MDSC/neutrophils (CD3^-^CD19^-^CD11c^-^CD11b^+^F4/80^-^Gr1^+^) and macrophages (CD3^-^CD19^-^CD11c^-^CD11b^+^F4/80^+^) is shown. **(C)** The percentage of H2Dd^+^ cells is shown for each type described in **(C**, **D)**. **(D)** Representative histograms for the expression of MHCII on myeloid-derived DCs. Data is representative of two independent experiments with n=3-5 per group (mean ± SEM). One-way ANOVA or Two-Way ANOVA was used to assess significance (*p<0.05, **p<0.01, ***p<0.001).

Similar to NK cells, the myeloid compartment was significantly enhanced by the treatment with UnLicNK cells (**Figures 1D** and **5**). An increase in the number of myeloid-derived DCs (CD3^-^CD19^-^CD11c^+^CD11b^+^), mainly from donor origin, was observed in the mice that received UnLicNK cells compared to IL-2 HCT and LicNK cells (**Figures 5B, C**). The DCs of this group also displayed a higher expression of MHCII (**Figure 5D**), which was particularly relevant in the donor-derived DC, suggesting a more mature phenotype. Allogeneic CD11b^-^ DCs (CD3^-^CD19^-^CD11c^+^CD11b^-^), monocytes, myeloid derived suppressor cells (MDSC) and neutrophils were also favored by the infusion of UnLicNK cells (**Figures 5B, C**). These results indicates that UnLicNK cells promoted both development and maturation of donor-derived myeloid cells, likely caused by the known ability of activated UnLicNK to secrete GM-CSF upon MHCI interaction, which is involved in myeloid cell differentiation (14).

**Figure 6:**
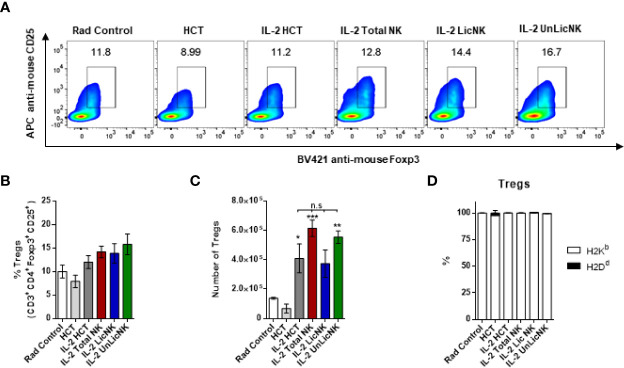
The number of Tregs is slightly increase by UnLicNK cell treatment during NMAC HCT. **(A)** Representative dot-plots of Foxp3 and CD25 is shown for gated CD4 T cells. **(B)** Total percentage of Foxp3^+^CD25^+^ CD4 Tregs is shown for gated CD4 T cells. **(C)** Total number of Tregs is shown. **(D)** Proportion of host (H2K^b^) and donor (H2D^d^) is shown for Tregs. Data is representative of two independent experiments with n=3–5 per group (mean ± SEM). Two-way Anova **(A)** or One-way ANOVA were used to assess significance. (*p<0.05, **p<0.01, ***p<0.001, n.s, no significance).

Regarding the B cell compartment, we observed an expansion of total and donor derived B cells after infusion of UnLicNK cells (**Figure 1**, **Supplementary Figure 5**). However, a decrease in the expression of the maturation marker IgM occurred after NK cell infusion, particularly in H2Dd^+^ B cells (**Supplementary Figure 5**) suggesting a stimulation of donor BM-derived B cell development with a more immature phenotype. A recent study demonstrated a role of Tregs in the maintenance of immune homeostasis and B cell differentiation (21). Thus, we next analyzed the presence of Tregs in the spleen at day 28 after NMAC HCT by flow cytometry Although the administration of total or UnLicNK cells did not significantly modified the percentage and total numbers of Tregs during NMAC HCT in IL-2 treated mice, more Tregs were actually observed in these two groups (**Figures 6A–C**). The majority of these Tregs were from host-origin (**Figure 6D**) like the rest of the T cell compartment.

### Infusion of Host-Derived Unlicensed NK Cells Results in a Tolerogenic Behavior Similar to the One Observed When Host-Derived Tregs Are Infused

**Figure 7:**
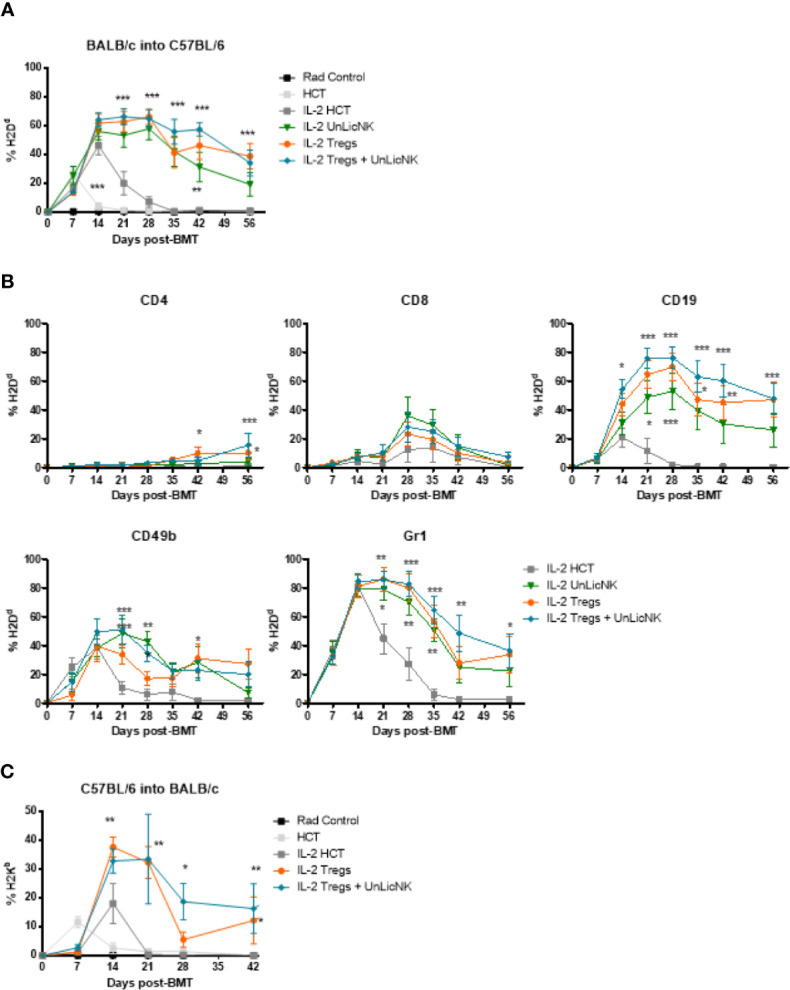
Immune reconstitution after infusion of host type UnLicNK cells and Tregs in NMAC allogeneic HCT. **(A)** Percentage of H2Dd^+^ donor cells after NMAC HCT is shown. **(B)** Percentage of H2Dd^+^ donor cells for gated CD4^+^, CD8^+^, CD19^+^, CD3^-^CD49b^+^, CD11c^+^, and Gr1^+^ cells is shown. **(C)** Percentage of H2K^b+^ donor cells after NMAC HCT is shown. Data is representative of three independent experiments with n=4–5 per group (mean ± SEM). One-way ANOVA or Two-Way ANOVA was used to assess significance. Significant differences are displayed for comparisons with the IL-2 HCT control group (*p<0.05, **p<0.01, ***p<0.001).

Previous studies have reported that administration of Tregs can achieve donor-specific tolerance and protect against GvHD (1, 5, 8–10, 22). Furthermore, exogenous administration of GM-CSF increases Tregs and ameliorates chronic GvHD through CD11c^+^CD8α^-^ DCs (23). Hence, enhanced tolerance and donor engraftment has also been observed when host-type Tregs are given during NMAC allogeneic HCT as well (8). These studies provide a rationale for combining host-type Tregs and UnLicNK cells into NMAC HCT to further improve donor chimerism. The infusion of UnLicNK cell and Tregs, alone or combined, caused a significant improvement of donor BM engraftment compared to the IL-2 HCT group, with a preferential increase of H2Dd^+^ B, NK and myeloid cells (**Figures 7A, B**). However, there were no differences in the magnitude and durability of engraftment between the groups that received these cells alone or combined (**Figure 7A**). Similar results were obtained when donor and host strains were exchanged (donor C57BL/6 and host BALB/c), suggesting that this effect is not strain-dependent (**Figure 7C**). It is important to note that the results obtained after UnLicNK cell infusion were very similar to those obtained by the tolerogenic immune Tregs, which therapeutic application in the clinic is limited by the low representation of this cell type (8).

## Discussion

In allogeneic HCT, reaching peripheral tolerance is still a pending issue. There has been many therapeutic strategies developed that aim to prevent alloreactivity against donor antigens, by directly targeting the cells involved in the alloreactivity or by promoting a more immunosuppressive environment (24). Some of these strategies do indeed involved adoptive cell therapy such as infusion of Tregs or NKT cells (1, 15, 24–27). Other immune cells that have also shown immune-tolerogenic properties in HCT are NK cells and MDSC (10, 12, 14, 24, 28). Within NMAC/RIC settings, however, new regimens to improve systemic immune tolerance across major histocompatibility barriers are also still necessary. Here, we demonstrate that the infusion of host-type UnLicNK cells is capable of increasing donor cell specific engraftment and achieves accelerated and durable mixed allogeneic chimeras.

We have shown that the infusion of host-derived UnLicNK cells alters the NK cell population by increasing the total number of NK cells, especially the ones derived from donor BMCs. It has been reported that UnLicNK cells can improve allogenic engraftment by the release of GM-CSF, a growth factor that is secreted by this particular subset when interacts with donor BMC during allogenic HCT in a SHP-1 dependent manner (14). GM-CSF is involved in hematopoiesis and its administration, combined with other cytokines, can accelerate hematopoietic recovery after allogeneic HCT (29, 30). Interestingly, long-term hematopoietic regeneration after syngeneic or allogeneic HCT was promoted by the treatment with a novel synthetic cytokine that was derived from the fusion of GM-CSF and IL-4 (31). Furthermore, during high-dose conditioning regimens, GM-CSF have shown to shorten neutropenia, a major cause of mortality in these settings (32). Accordingly, higher levels of GM-CSF were detected in the serum of mice treated with total or UnLicNK cells during NMAC HCT. In is important to note that the high frequency of UnLicNK cells presence in the composition of *ex vivo* IL-2 expanded total NK cells (14, 18, 33) contribute to the similarities observed between the infusion of UnLicNK and total NK cells in some of the parameters analyzed.

NK cells can also regulate donor cell reconstitution by directly modulating reactive T cells or antigen presenting cells in a NKG2D-, FasL-, IL-10-, or perforin-dependent manner (10, 11, 16, 34). TGFβ has been also attributed a part in the immunosuppressor function of NK cells (35), but no differences in the secretion of TGFβ were found between sorted LicNK and UnLicNK cells and will unlikely play a role in our model. Taking in consideration our results and these studies, NK cells, in our scenario, could modulate alloengraftment by exerting both an immunosuppressive (regulate T cells) and a graft supporting effect (secrete GM-CSF).

Additionally, the early reconstitution and expansion of donor NK cells, which functional capacities are maintained, along with the effect of infused UnLicNK cells themselves, could potentially lead to a stronger and earlier protection against cancer relapse and opportunistic infections. Accelerate NK cell reconstitution and expansion during HCT is expected to enhanced response against cancer and viral infection when immunotherapies targeting NK cells are applied (36–38). Such is the case for IL-2 and anti-TGFβ combinatorial therapy (39, 40). Additionally, adoptive NK cell transfer therapies, and lately CAR-NK cell therapy, have proven effective in hematological malignances (8, 12, 41–43). If we focus more on NK cell subsets, it has also been shown that UnLicNK cells in HLA-matched HCT after myeloablative therapy are functionally competent against tumors expressing self-HLA immediately after transplantation unlike LicNK cells, demonstrating that alloreactivity can be obtained with HLA-matched donor NK cells by selecting those NK cells that express inhibitory receptors for non-self HLA (44). In line with these data, an increase of IFNγ secretion was observed in cultures of sorted UnLicNK cells with cells expressing self-MHCI when compared to LicNK cells. Moreover, a previous study have shown that the lysis of YB tumors transfected with self-HMC (YB-H2D^b^) was also enhanced in H2^b^-derived UnLicNK cells when compared to LicNK cells, whereas no differences were found against tumors expressing non-self-MHCI (YB-H2D^d^) (33). Similarly, a rapid reconstitution of NK cells with inhibitory receptors for non-self HLA has been correlated with good prognosis in neuroblastoma, non-Hodgkin’s lymphoma, Hodgkin’s disease, acute myeloid leukemia, multiple myeloma, and metastatic breast cancer (45). UnLicNK cells were also involved in the anti-tumor efficacy of anti- disialoganglioside GD2 monoclonal antibody therapy for high-risk neuroblastoma patients (46). Additionally, we observed that infusion of UnLicNK cells induce an increase of CD8 T cells with a memory phenotype and with their proliferative abilities improved, while their functional capacities remain intact. It has been reported that donor CD8^+^CD44^high^ memory T cells have a protective effect against GvL without causing GvHD (31). Likewise, homeostatic reconstitution from a lymphopenic stage in sublethally irradiated mice have showed a greater expansion of this particular subset and was correlated with protection against tumor growth (20). Although evaluating the anti-tumor efficacy of this therapy is out of the scope of this study, all these studies suggest that the adoptive transfer of host-derived UnLicNK cells could potentially help not only towards tolerance of allogeneic cells in HCT, as we report here, but also protect from cancer. Therefore, further analysis to evaluate the anti-tumor efficacy of UnLicNK cells during NMAC HCT is necessary.

The importance of MDSC in solid organ and HCT has been highlighted in recent studies due to their potential role in immune tolerance (28, 47–49). An early recovery of MDSC has been positively correlated with enhanced tolerance in 26 patients undergoing allogeneic HCT (50). In this study, tolerance was attributed to the suppression of third-party CD4 T cell proliferation as well as Th1 differentiation. A higher presence of Tregs was also reported in those patients (50). Other studies suggest alternative mechanisms such as a strengthened crosstalk between MDSCs and Tregs or NKT cells (28, 51). In agreement with these studies, an increase of the myeloid cell compartment was also observed at day 28 post-HCT after treatment with UnLicNK cells, but an increase on CD4 T cells or NKT cells was not observed at this time point in the organs analyzed.

Increasing the presence of Tregs by cell transfer therapy or targeting its expansion, are therapeutic strategies highly explored during allogeneic HCT settings with promising results (7, 9, 26, 27, 52). However, there are still some limitations surrounded Tregs adoptive transfer therapy that mainly fall into two categories, the low proliferative rate of this cell type and its paucity, which limit the cell numbers that can be obtained for cell therapy to achieve biological effects (17). A study performed by Hotta and collaborators suggested that GM-CSF therapy could mitigate GvHD by promoting Tregs proliferation (53). In our model, host-derived Tregs were expanded in all groups that received IL-2, as it was expected given the role of IL-2 in the preferential expansion of Tregs due to their expression of the high affinity IL-2Rα, and no significant changes were found between these groups. However, a tendency towards higher numbers of Tregs was observed in the mice that received Total or UnLicNK cells. Therefore, our intention for combining Tregs and NK cell transfer during NMAC allogeneic HCT was to obtain an additive or synergistic tolerogenic effect with highly sustainable donor-host chimeras that will allow for the infusion of lower doses of Tregs. Unfortunately, the combination of both cell types did not improve donor cell engraftment, even when a high therapeutic dosage of Tregs was given (27). It is possible that the co-administration of Tregs and UnLicNK did not cause an additive/synergistic effect because the exogenous administration of Tregs bypassed the need for UnLicNK cells. Still, it is important to note that we did achieve a similar level of chimerism between the groups that received UnLicNK cell and Tregs cell therapy. Because obtaining large numbers of NK cells for therapeutic usage is more attainable, these results advocate for UnLicNK cell adoptive transfer therapy as a promising therapeutic alternative to Tregs to promote donor chimerism during NMAC settings. Furthermore, the use of NK cells to improve allogeneic engraftment represents other advantages given the versatility to manipulate (by cell sorting or neutralizing antibodies against KIRs) and expand the NK cell subset of interest. Consequently, these results offer evidence for the potential therapeutic use of UnLicNK cells in HCT to give a much-needed upgrade to the NMAC regimen protocols.

## Data Availability Statement

The raw data supporting the conclusions of this article will be made available by the authors, without undue reservation.

## Ethics Statement

All animal studies and protocols were reviewed and approved by the IACUC at Stanford University.

## Author Contributions

MA designed and performed the research, analyzed the data, and wrote the manuscript. AP, FS, JB, and KM-B. contributed in conducting the experiments. AP, FS, JB, KM-B, and TH provided scientific input and assisted with the preparation of the manuscript. RN provided overall scientific guidance and helped write the manuscript. All authors contributed to the article and approved the submitted version.

## Funding

This work was supported by the National Institute of Health grants RO1CA125276 and P01CA049605. MA was supported by the AACR-Millennium Fellowship in Lymphoma Research (15-40-38-ALVA), the ASBMT New Investigator Award, the Marie Skłodowska-Curie fellowship (CINK 746985), and by the Spanish Association Against Cancer’s Investigator grant (2019 AECC Investigator). AP was supported by the ASBMT New Investigator Award. FS was supported by the Geneva University Hospitals, the Swiss Cancer League, the Fondation Genevoise de bienfaisance Valeria Rossi di Montelera and the Dubois-Ferrière-Dinu-Lipatti Foundation. KM-B was supported by the German Cancer Aid. The FACSAria II (BD Bioscience, San Jose) used in this project was obtained through the grant S10RR025518-01.

## Conflict of Interest

The authors declare that the research was conducted in the absence of any commercial or financial relationships that could be construed as a potential conflict of interest.
