# Supplementary material for: Infusion of Host-Derived Unlicensed NK Cells Improves Donor Engraftment in Non-Myeloablative Allogeneic Hematopoietic Cell Transplantation
Source: Front Immunol. 2021 Jan 7;11:614250. doi: 10.3389/fimmu.2020.614250 (PMC7817981; doi:10.3389/fimmu.2020.614250)
Supplement: Supplementary file 1 [file DataSheet_1.pdf]

Antibody	Clone	Provider	Catalogue
CD3	145-2C11	BioLegend	100334, 100355
CD11b	M1/70	BioLegend	101259, 101208
CD11c	N418	BioLegend	117334
CD19	6D5	BioLegend	152404, 115541
CD25	PC61	BD Bioscience	565314
CD4	RM4-5	BioLegend	100552
CD4	GK1.5	BD Bioscience	612952
CD44	IM7	BioLegend	103006
CD45.1	A20	BioLegend	110724, 110730, 110736
CD45.2	104	BioLegend	109820, 109827, 109835
CD49b	DX5	BioLegend	108906
CD62L	MEL-14	BioLegend	104448
CD8	53-6.7	BioLegend	100714, 100744
CD8	53-6.7	BD Bioscience	563795
Eomes	Dan11mag	Thermo Fisher Scientific	50-4875-80
Foxp3	MF-14	BioLegend	126419
H2Kb	SF1-1.1	BioLegend	116506, 116515, 116618
H2Dd	34-2-12	BioLegend	110608, 110612
I-A/I-E	M5/114.15.2	BioLegend	107614
IgM	RMM-1	BioLegend	406508
IFNg	XMG1.2	BioLegend	505814, 505838
Ki67	16A8	BioLegend	652420
KLRG1	2F1/KL1261	BioLegend	138409, 138423, 138414
Ly49C/I	5E6	BD Bioscience	557418
Ly49G2	4D11	BD Bioscience	555315, 742885
Ly49G	4T8	Thermo Fisher Scientific	13-5885-82
Ly6C/Ly6C Gr1	RB6-8C5	BioLegend	108416
Ly6G	1A8	BioLegend	127633
Mouse IgG1, Kappa		Thermo Fisher Scientific	50-4714-80
NK1.1	PK136	BioLegend	108720, 108722, 108745, 108748
Rat IgG1, Kappa	RTK2071	BioLegend	400418, 400443
Streptavidin		Thermo Fisher Scientific	46-4317-82
Streptavidin		BioLegend	405229
T-bet	4B10	BioLegend	25-5825-80
TCRb	H57-597	BioLegend	109243, 109226, 109241
Thy1.2	30-H12	BioLegend	105324, 105331, 105343

Supplemental Table 1. Antibodies for flow cytometry. The table lists all antibodies used in this study, including clone (when applicable), provider and catalogue number.

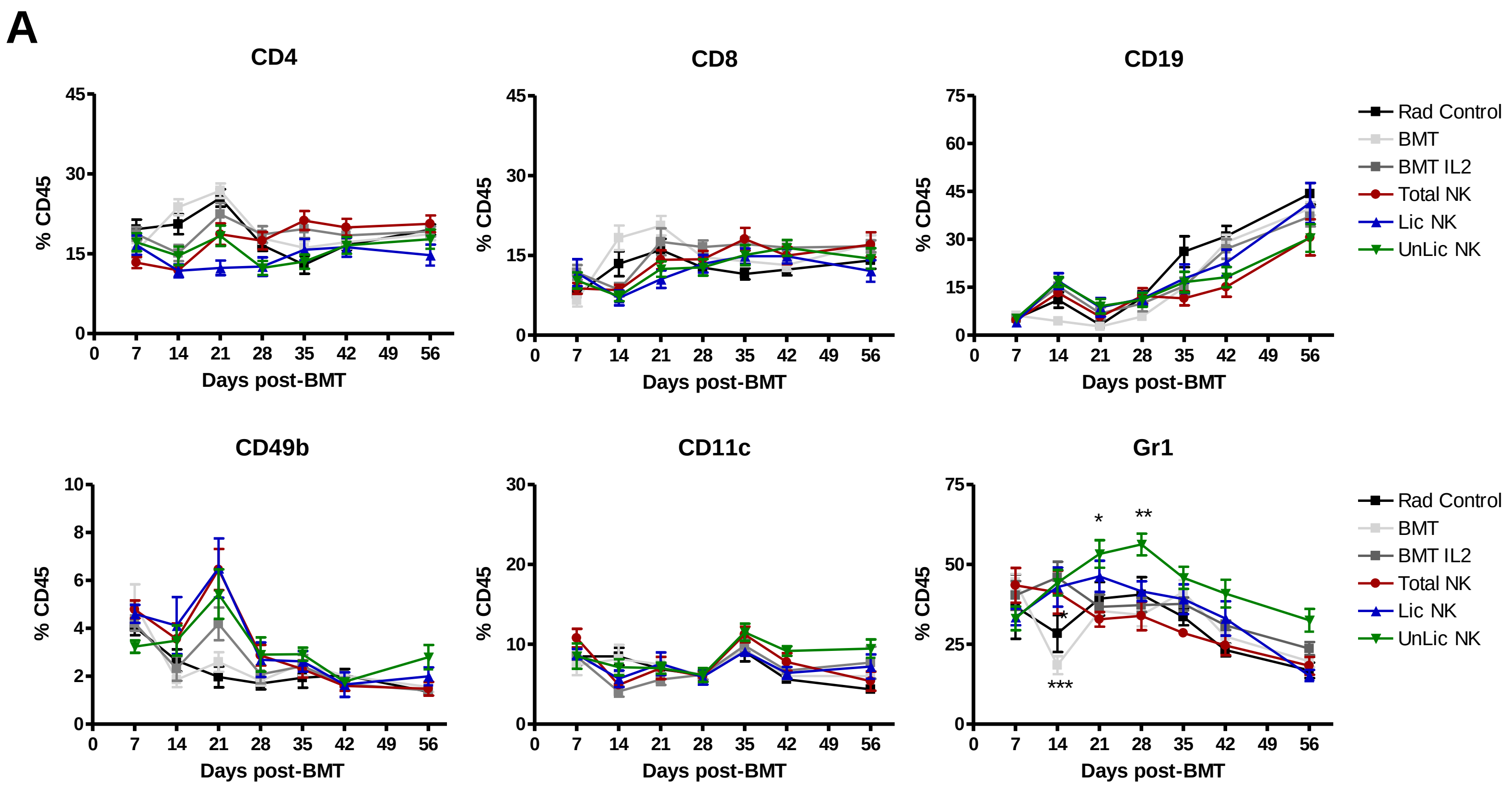

Supplemental Figure 1. Administration of ex vivo activated UnLicNK cells causes the myeloid compartment frequency to increase during NMAC HCT. (A) The changes in CD4, CD8, CD19, NK, DC and myeloid cells is shown in the PB of gated CD45.2^{+} cells after NMAC HCT. Data is representative of two independent experiments with n=3-5 per group (mean ± SEM). Two-way ANOVA was used to assess significance. (*p<0.05, **p<0.01, ***p<0.001, n.s. no significance).

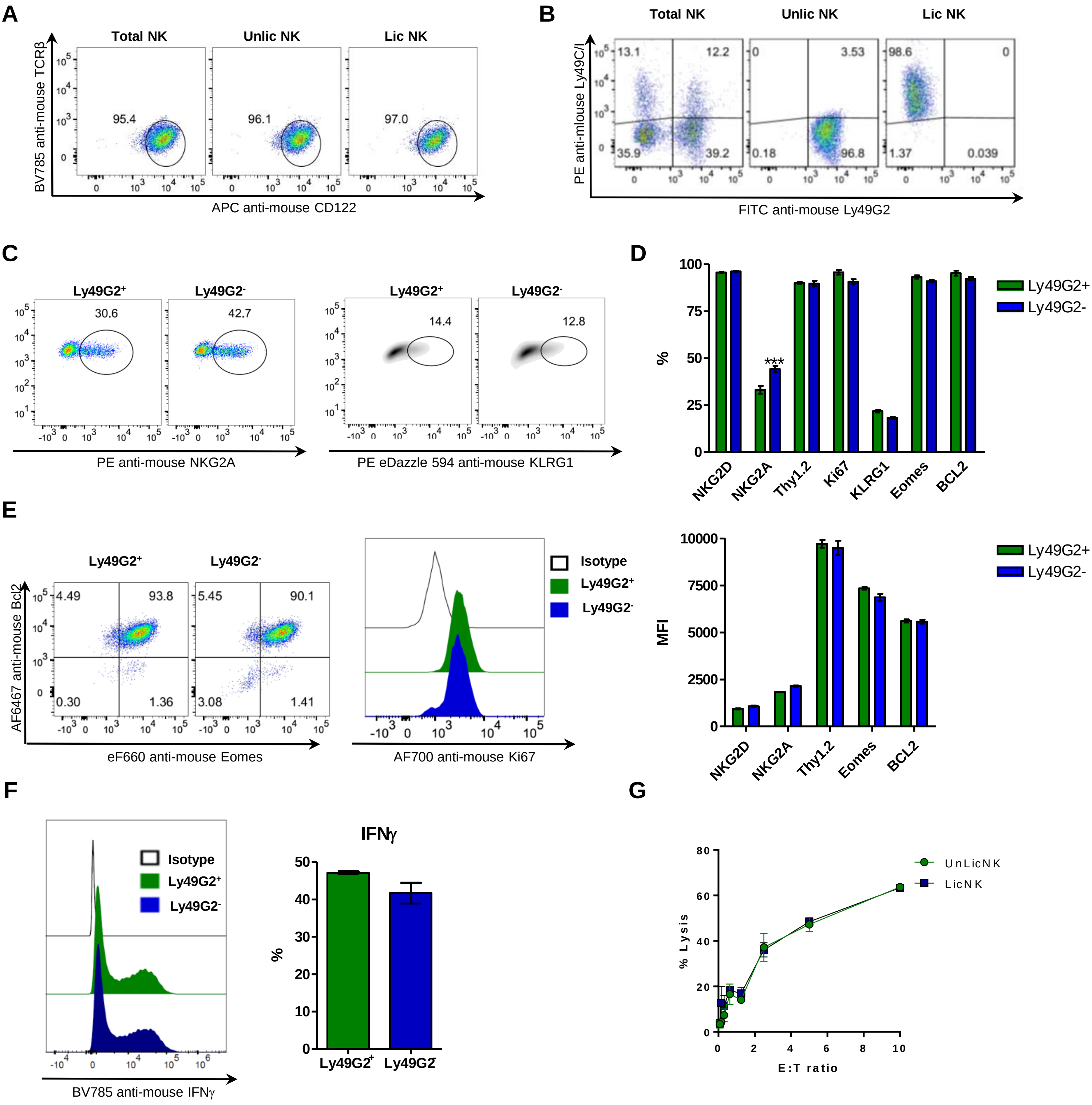

Supplemental Figure 2. Differential expression of NK cell activation markers on activated NK cell subsets. Adherent NK cells were collected after 5 days of in vitro IL-2 culture and stained for CD3, CD122, Ly49C/I and Ly49G2 followed by cell sorting. **(A-B)** Representative dot-plots of sorted NK (CD45.1$^+$TCRb$^-$CD122$^+$Ly49C/I$^{+/-}$ Ly49G2$^{+/-}$), UnLicNK (CD45.1$^+$TCRb$^-$CD122$^+$Ly49C/I$^-$Ly49G2$^+$) and LicNK (CD45.1$^+$TCRb$^-$CD122$^+$Ly49C/I$^+$Ly49G2$^-$) cells prior to infusion in host mice. **(C)** Representative density-plots of NKG2A and KLRG1 expression of gated Ly49G2$^+$ and Ly49G2$^-$ NK cells. **(D)** Total percentage (upper panel) and MFI (lower panel) of NKG2D, NKG2A, Thy1.2, Ki67, KLRG1, Eomesodermin (eomes) and BCL2 of gated Ly49G2$^+$ and Ly49G2$^-$ NK cells are shown. **(E)** Representative dot-plots of BLC2 and Eomes distribution, and Ki67 expression is shown for gated Ly49G2$^+$ and Ly49G2$^-$ NK cells. **(F)** Representative histogram and total percentage of IFNγ producing cells upon NK1.1 stimulation on gated Ly49G2$^+$ and Ly49G2$^-$ NK cells is shown. **(G)** Percentage of lysis for a 4h standard Cr-release assay against Yac-1. Data is representative of four independent experiments with n=3 per group (mean ± SEM). Two-way ANOVA or student t-test was used to assess significance (***p<0.001).

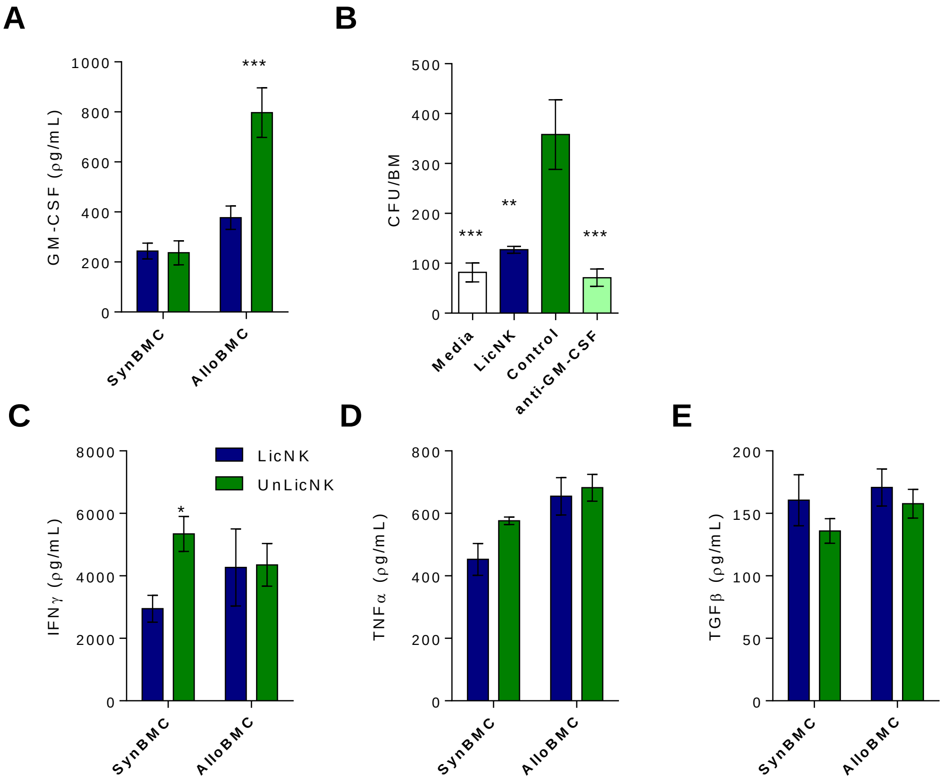

Supplemental Figure 3. Role of GM-CSF in the effect of UnLicNK cells to favor alloengrafment. Sorted LicNK or UnLicNK were co-cultured with syngeneic (SynBMC) or allogeneic (AlloBMC) for 24h. Supernatants were collected to asses cytokine levels and BMC were later cultured in a CFU-c assay. In some experiments 50μg/mL anti-GM-CSF were used to block GM-CSF. **(A)** Levels of GM-CSF detected on the supernatant of in vitro cultures from sorted LicNK or UnLicNK with synBMC or alloBMC. **(B)** The hematopoietic content (CFU) of alloBMC previously cultured for 24h with media only, LicNK or UnLicNK (control) with or without anti-GM-CSF or SSG treatment. **(C-E)** Levels of IFNγ (C), TNFα (D) and TGFβ (E). Data represents one experiment done in triplicate. One-way or two-way Anova were used to assess significance. (*p<0.05, **p<0.01, ***p<0.001, n.s. no significance).

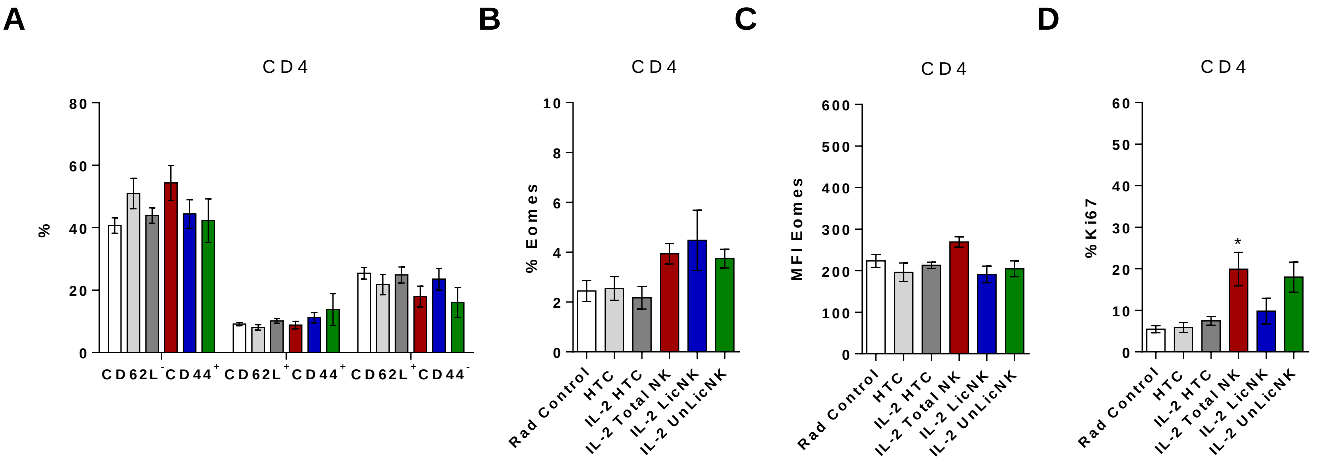

Supplemental Figure 4. Impact of UnLicNK cell infusion in the CD4 T cell compartment. (A) The distribution of effector memory (CD62L$^-$CD44$^+$), central memory (CD62L$^+$CD44$^+$), and naïve (CD62L$^+$CD44$^-$) cell subsets for CD3$^+$CD4$^+$ T cells is shown. **(B)** The percentage of eomes is shown for gated CD4 T cells. **(C)** The MFI of eomes is shown for Eomes$^+$ CD4 T cells. **(D)** Percentage of Ki67 on gated CD4 T cells. Data is representative of two independent experiments with n=3-5 per group (mean ± SEM). Two-way Anova (A) or One-way ANOVA were used to assess significance. (*$p<0.05$, **$p<0.01$, ***$p<0.001$, n.s. no significance).

Supplemental Figure 3

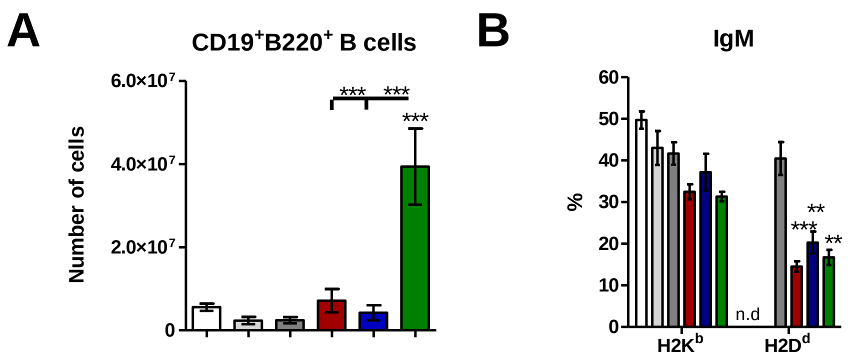

Supplemental Figure 5. UnLicNK cells promotes the development of immature B cells after NMAC HCT. (A) Total number of $CD19^{+}B220^{+}$ B cells is shown. **(B)** The expression of IgM for $H2Kb^{+}$ and $H2Dd^{+}$ B cells is shown. Data is representative of two independent experiments with n=3-5 per group (mean ± SEM). One-way ANOVA was used to assess significance. (*p<0.05, **p<0.01, ***p<0.001, n.s. no significance).
